# The Production of Relatives in Mandarin Children With Specific Language Impairment–From the Perspective of Edge Feature Underspecification Hypothesis

**DOI:** 10.3389/fpsyg.2021.705526

**Published:** 2021-10-04

**Authors:** Haiyan Wang, Haopeng Yu

**Affiliations:** ^1^School of Foreign Languages, Xinxiang Medical University, Xinxiang, China; ^2^Faculty of International Studies, Henan Normal University, Xinxiang, China

**Keywords:** relative clauses, production, asymmetry, specific language impairment, Edge Feature Underspecification Hypothesis

## Abstract

This paper is a first attempt to investigate the production of Relative Clauses (RCs) in Mandarin children with Specific Language Impairment (SLI) (aged 4; 5 to 6; 0) and their typically developing (TD) peers. The data from a preference choice task suggested that (i) Children with SLI performed better on the subject-gapped than object-gapped RC; (ii) Children with SLI performed substantially worse than their TD peers on the RCs production; (iii) Children with SLI were more inclined to omitting the complementizer and using simple sentences and sentence fragments as avoidance strategies. The Edge Feature Underspecification Hypothesis may explain not only the asymmetry of production seen in children with SLI, but also the presence of errors and avoidance strategies used by this population in the task.

## Introduction

Almost all studies on languages with head-initial RCs found a preference for subject RCs in production, whereas the results on the production of Mandarin RCs are inconsistent: a primacy for subject RCs, object RCs or no asymmetry in the production (e.g., Su, 2006; Hsu et al., 2009; Chen and Shirai, 2015; Hu et al., 2015). Additionally, to date, there are few studies that investigated the production of RCs in Mandarin children with SLI. The first focus of this paper is to establish whether there is a primacy for subject-gapped RCs (subject RCs) or object-gapped RCs (object RCs) production in this population.

It is now well acknowledged from a variety of studies that children with SLI have problems with the production of RCs (e.g., Novogrodsky and Friedmann, 2006; Jensen de López et al., 2014; Adani et al., 2016). However, there has been little agreement on what constitutes the underlying cause of these problems. Furthermore, research on the topic has been mostly restricted to explanation of the primacy for subject RCs but failed to address the nature of errors observed in children with SLI, such as the omission of complementizers. In this paper, we propose the Edge Feature Underspecification Hypothesis (EFUH; Yu, 2018; Yu et al., in press) to provide a better explanation of syntactic deficit in children with SLI.

This paper has been organized in the following ways. The first section begins by briefly reviewing previous studies on the production of RCs in children with and without SLI, followed by laying out the theoretical issues of SLI. It then goes on to present the elicitation production experiment and the results. The last section deals with the discussion and concluding remarks.

To have a better understanding of RC production in children with SLI, a brief review of the studies on the acquisition of RCs in TD children is warranted. Extensive research on RC acquisition in many languages indicates that children younger than 6 acquire subject RCs with greater easiness than object RCs, such as in English (Zukowski et al., 2009); German (Adani et al., 2013); Hebrew (Arnon, 2010); Italian (Guasti and Cardinaletti, 2003; Contemori and Belletti, 2014) and Tagalog (Tanaka et al., 2019). Various explanations have been put forward to account for the findings, which can be roughly divided into the usage-based approach, the processing account based on linear word order and the account based on syntactic structure. Unfortunately, the three accounts usually converge in languages with head-initial RCs, such as English, all pointing to the subject over object RCs advantage.

Under the multifactorial usage-based account (Diessel and Tomasello, 2005; Diessel, 2007), children acquire RCs in a piecemeal fashion by producing new RC constructions based on simpler constructions, which have been deeply entrenched in their minds. As the examples in (1) illustrate, English subject RCs maintain the canonical subject–verb–object order of English, whereas English object RCs have an object-subject-verb order, which is different from the basic word order in English. It is the canonical word order in subject RCs that facilitates the production of this kind of RCs according to this account. In the same vein, Arnon (2010) suggests that mastery of RCs emerges as a gradual expansion of uses, consistent with usage-based account.


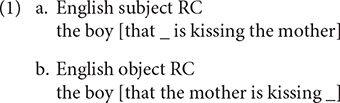


The processing based account also correctly predicts the subject over object RC advantage in English. Hawkins (2004; p. 173) proposed that filler–gap dependency places a burden on processing resources. In order to integrate a filler (the relative head in the case of RCs) into the sentence, the human processor needs to maintain the filler in the working memory until the processor encounters a gap, at which time the dependency between the filler and the gap can be established. As a consequence, RCs with greater linear distance between a gap and its filler are harder to process, because such RCs tax more on the processor. The account quantifies the distance between the gap and the filler in terms of the number of elements with discourse referents (essentially, nouns, and verbs) occurring between the filler and the gap (Gibson, 1998, 2000; Warren and Gibson, 2002; Grodner and Gibson, 2005; p. 252; Lewis et al., 2006). The establishment of filler–gap dependency in the subject RC (1a) is less demanding for working memory, because no element with discourse referents intervenes between the filler and the gap. On the contrary, there are two elements with discourse referents (the NP *the mother* and the verb *kiss*) intervening between the filler and the gap in the object RC (1b).

Nevertheless, the subject-object RC asymmetry can also be explained by theories capitalizing on structural distance between the filler and gap. O’Grady (1997) suggests that the structure with more deeply embedded gaps is more complex and thus more difficult for children to produce. A structure’s complexity increases with the number of XP categories intervening between the gap and the filler (O’Grady, 1997; p. 136). Along similar lines, Hawkins (1999) proposes the Minimal Distance Hypothesis, in which the filler-gap distance is measured on the basis of the structural hierarchy. The distance between the filler and the gap is determined by counting all the non-terminal nodes and terminal nodes in the hierarchical structure. In line with such theories, children encounter greater difficulties in producing English object RCs, in which the gap is more deeply embedded, as illustrated in (2), because the object RC (2b) is more complex. In (2b), three XP categories (CP, IP, and VP) intervene between the gap and the head noun, whereas two XP categories (CP and IP) occur between the gap and the head noun in the subject RC (2a).


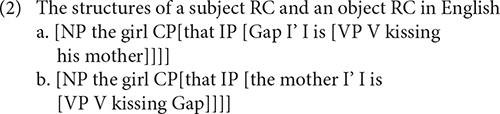


The theories presented thus far all explain the subject RCs primacy in the acquisition of English, although the first two accounts capitalize on the surface word order, and the last one on the hierarchical structure. Because of this, the surface word order and structural factors can not be teased apart in head-initial RCs (e.g., English RCs). Mandarin is a typologically rare language with the SVO main clause and the head-final RCs, as in (3). The surface word order and structural factors pull in opposing directions: the surface word order favors the object over subject RCs advantage, whereas the structural factor favors the opposite, so the investigation of the acquisition of Mandarin RCs is helpful to establish which factor counts more.


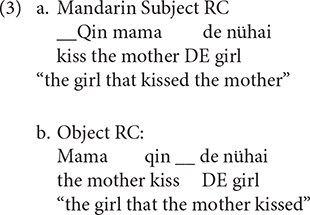


However, previous production studies of Mandarin RCs in TD children yielded inconsistent results. In total, there are three views on the sequence of Mandarin RCs acquisition: subject RCs advantage, object RCs advantage and no asymmetry.

Data from several studies suggests that there is a subject RC preference in the production by Mandarin-speaking children, which is consistent with the pattern found in languages with head-initial RCs. In an elicited production task modeled after Hamburger and Crain (1982), Hsu et al. (2009) tested 23 Mandarin-speaking children (with a mean age of 4;8) and 10 adults and found a clear subject RCs advantage in children irrespective of the embedding context of RCs. Likewise, Hu et al. (2015) found a subject RC preference in children of all ages in a study investigating the production of RCs in 125 Mandarin children (aged 3;0 to 8;11) by using a preference choice task modeled after Novogrodsky and Friedmann (2006). They captured the subject-object RCs asymmetry under the Relativized Minimality (RM; Rizzi, 1990, 2004). Precisely, in subject RCs, no structural intervener occurs between the relative head and its copy, whereas in object RCs, a qualified element (the embedded subject) intervenes between them, thereby causing more difficulty in object RC production.

However, some materials were arguably problematic in both Hsu et al. (2009) and Hu et al. (2015). For example, as noted by Hu et al. (2015), in Hsu et al. (2009), some verbs in the subject RCs could be used intransitively (e.g., *nage nühai zai huahua/changge* “That girl is drawing/singing”), whereas all verbs are transitive in object RCs. The verb choice in the subject RCs condition may inflate the subject RCs advantage. The materials used in Hu et al. (2015) are not exempt from criticism. In the study, three verbs (*he* “drink,” *chi* “eat,” *mai* “buy”) are problematic in subject RCs condition, because only semantically irreversible RCs might be built on them. Such RCs are less demanding on children since they can be interpreted without requiring full syntactic processing (Kim and O’Grady, 2016). On the other hand, all the stimuli are semantically reversible in object RCs condition. Because of this, the choice of verbs in this study may also broaden the gap between the subject and object RCs.

Other studies suggest that Mandarin object RCs are acquired earlier and with greater ease. Chen and Shirai (2015) discovered a primacy of object RCs in early child speech by analyzing the spontaneous production of four Mandarin-speaking children (aged 0;11--3;5)^1^. They claimed that the preference for object RCs is due to the fact that Mandarin object RCs exhibit the same typical SVO word order as canonical simple sentences. Furthermore, they also argued that the object RCs advantage might also be influenced by the frequency of input, because they found that object RCs were more frequently used than subject RCs in parental speech during interactions between children and their caregivers.

Although Chen and Shirai’s (2015) study revealed the early trajectory of Mandarin children’s RCs production, their claim has been strongly contested by a number of researchers. First, Hu et al. (2015) argued that the object RCs advantage was established merely on a numerical basis. Second, the object RC primacy found in parental speech is at odds with the results from several corpus studies, which consistently revealed a subject RC advantage in Mandarin-speaking adults (Hsiao and Gibson, 2003; Pu, 2007; Wu et al., 2010). Third, as shown in Hsu (2014), the majority of object RCs reported in Chen and Shirai (2015) are associated with the so-called “cleft construction,” which typically puts a particular constituent into focus, as illustrated in (4). Children tended to omit the focus marker (*SHI*) and drop the head, which accounts for the overwhelmingly large number of the headless object RCs found in the corpus.


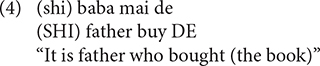


The third view regarding this issue is that there is no asymmetry in the production of subject and object RCs by Mandarin-speaking TD children. Su (2006) tested two groups of children (5;0 to 6;5) and 31 adults by adopting the elicitation method in Hamburger and Crain (1982) with minor revision. The results revealed that there was no difference between the subject and object RCs production in either the two groups of children or adults. As noted by Hu et al. (2015), Su’s results should be interpreted with caution because they were obtained with two trials for each sentence type. Thornton (1996) recommends using at least four tokens of each sentence type in the elicited production task.

Furthermore, all of the studies reviewed thus far have one flaw in the coding system. According to Liu (2005), the demonstrative pronoun (*ne*) can be used as a relative marker in Mandarin, as shown in (5).

^2^ 
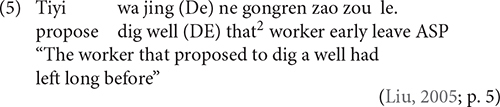


The first reason for this assumption is that with the presence of the demonstrative pronoun, the normal relative marker DE, usually preceding the demonstrative pronoun, can be omitted, as shown in (5). The second is that when the demonstrative is deleted, the relative marker must be added; otherwise, the sentence is ungrammatical, as demonstrated in (6).


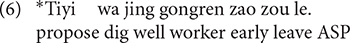


If we accept this assumption, RCs with demonstrative as the relative marker should be categorized as the target responses in the coding system; otherwise we risk underestimating the RC production in Mandarin children.

To summarize, it is difficult to interpret the results of the aforementioned studies because of the problematic design and coding system. Additionally, although extensive research has been carried out on the acquisition of RCs in Mandarin TD children, no single study exists which investigates the acquisition of RCs in Mandarin children with SLI. We can hardly evaluate which preference holds in Mandarin RCs acquisition in this population. In this paper, we will test the production of semantically reversible RCs and improve the coding system, which may provide a more comprehensive understanding of the production of RCs in Mandarin children with SLI.

Being similar to the studies on the acquisition of RCs in TD children, studies on children with SLI also concentrate on the question of which RC is preferred in the production. Furthermore, the researchers showed even greater interest in the question of what factors contribute to the decayed ability to produce RCs in this group of children, namely, the relationship between syntactic impairment and the production of RCs.

There is now a substantial amount of data amassed cross-linguistically on RCs production in children with SLI, revealing that they exhibit severe deficits in the production of RCs across typologically different languages, such as English (Schuele and Nicholls, 2000; Schuele and Tolberrt, 2001; van der Lely and Battell, 2003; Schuele and Dykes, 2005; Frizelle and Fletcher, 2014), Swedish (Håkansson and Hansson, 2000), Hebrew (Novogrodsky and Friedmann, 2006), Italian (Contemori and Garraffa, 2010; Garraffa et al., 2015), Greek (Stavrakaki, 2001, 2002), Danish (Jensen de López et al., 2014), and German (Adani et al., 2016). Nonetheless, the results of the previous studies vary depending on the age of the children (Novogrodsky and Friedmann, 2006) and on the elicitation tasks adopted. Many theories have been proposed to explain the source of difficulty seen in children with SLI when producing RCs.

The first account attributes the poor performance of producing RCs in children with SLI to the impaired knowledge concerning syntactic movement. Some researchers maintained that syntactic movement is inaccessible to children with SLI, whereas others argued that children with SLI do possess the knowledge of syntactic movement, albeit it is difficult for them to achieve.

The difficulty of producing RCs in preschool children with SLI was attributed to their inability to project a fully fledged clause structure. It has been shown that children with SLI tend to omit the obligatory complementizer and there is a 2-year delay in the onset of RCs production in this population (Håkansson and Hansson, 2000; Schuele and Nicholls, 2000; Schuele and Tolberrt, 2001; Schuele and Dykes, 2005). Håkansson and Hansson (2000) proposed that children with SLI have difficulties with functional categories, including complementizers, thereby resulting in problems with projecting a fully fledged CP. Schuele and Nicholls (2000) reported that when English-speaking children with SLI begin to attempt subject RCs, they tend to omit the obligatory complementizers, suggesting there are linguistic vulnerabilities concerning functional categories and they produce subject RCs as late as 5 or 6 years of age, indicating the delayed emergence of RCs. They maintained that the results are consistent with Leonard’s (1995) functional category deficits account, which implies that the absence of the compulsory complementizer indicates that the underlying grammar has a poorer representation of functional categories.

Stavrakaki (2002) tested Greek-speaking children with SLI (aged 5–9) using a toy elicitation task (Crain and Thornton, 1998) and found that the overall accuracy of the RCs production in TD children was far greater than that in children with SLI. Simple active sentences, coordinated structures and RCs with missing heads are included in the avoidance strategies employed by children with SLI. The researcher maintained that headless RCs indicate that co-indexation between the variable bound by the operator in (Spec, CP) and the head can not be established, which in turn proves the absence of the operator movement^3^. He further argued that decay in production of RCs suggests that the knowledge of relativization is absent in the grammar of children with SLI, notwithstanding the production of few target-like RCs.

Contemori and Garraffa (2010) found that in both subject and object RCs elicitation tasks, Italian children with SLI (aged 4;5 to 5;9) performed more poorly than TD children. In the majority of cases where an RC was targeted, children with SLI either gave “no response” or produced a declarative clause. Furthermore, they concluded that the lack of the complementizer is the most commonly attested atypical production in children with SLI, indicating the absence of a CP layer in the grammar of this population.

To sum up, all the studies listed above converge in terms of the source of difficulty shown in RCs production, all pointing to the absence of the relativization in the narrow syntax of children with SLI. However, one of the limitations of this explanation is that it does not explain why children with SLI can produce target-like RCs if the syntactic movement is inaccessible to them.

Other critics have countered the above argument by claiming that the *Wh*-movement is difficult for children with SLI, but not entirely absent or optional in this population (e.g., Adani et al., 2016). The researchers examined the production of RCs in German-speaking children with SLI (4;7–10;11) and discovered that notwithstanding substantial difficulty with producing RCs, children with SLI did demonstrate some ability to produce adult-like embedded sentences derived by *Wh*-movement, which contradicts the assumption that the *Wh*-movement is absent in children with SLI. At the same time, their results revealed that children with SLI produced very few instances of filled object gap structures (RCs with *in situ* heads), which is in contrast to the Representational Deficit for Dependent Relationship theory (RDDR; van der Lely and Battell, 2003), which proposes that *Wh*-movement is wrongly marked as optional in the grammar of children with SLI.

The second group of researchers considers that the deficiency in children with SLI is due to thematic role assignment to displaced constituents rather than impaired knowledge of syntactic movement (Novogrodsky and Friedmann, 2006; Jensen de López et al., 2014).

Novogrodsky and Friedmann (2006) examined the production of RCs in 18 Hebrew-speaking children with SLI (9;3–14;6) and 28 TD children (7;5–11;0) by using a preference task and a picture description task. The researchers found that children with SLI performed significantly worse than TD participants and they were more accurate on subject RCs than object RCs. The most common errors detected in children with SLI in the object RC condition were thematic errors and reduction of thematic roles (18%), no movement from object position (8%), and production of simple sentences instead of RCs (14%). Most notably, they found that children with SLI did not omit complementizer. They concluded that the deficit in children with SLI is primarily related to thematic role assignment to displaced constituents, rather than a structural deficit in embedding.

Jensen de López et al. (2014) explored the production of subject and object RCs in 18 Danish-speaking children with SLI (5;0 to 8;4) and discovered that children with SLI performed less well than two groups of TD children in producing the subject RC, while no significant difference was detected between the three groups in the production of object RCs. A preference for subject RCs was found in all the three groups, which has been attributed to the RM effect involved in the object RC following Friedmann et al. (2009). Simple sentences were the predominant error type in children with SLI in the subject RC task, but in the object RC task, they opted for simple sentences, passive object relatives, fragments and thematic role reversal errors when they could not produce targeted RCs. They did not find any difference between the three groups of children in omitting the obligatory complementizer. Following Novogrodsky and Friedmann (2006), they argued that the deficiency in children with SLI is linked to the assignment of thematic roles.

While both the two studies found that children with SLI have impaired ability of thematic role assignment, this inability is not limited to them, as TD children also have made thematic role reversal errors, implying that this deficit does not reflect the qualitative difference between children with and without SLI. Furthermore, this line of reasoning does not sufficiently account for the inability to project a fully fledged clause structure seen in preschool children with SLI.

Recently, Corver et al. (2012) and Lorenzo and Vares (2017, 2019) proposed that the syntax-phonology interface, specifically the externalization channel, was pinpointed as the primary locus of affectation of SLI. According to Corver et al. (2012), the language problem of this population appears to be the mapping of an adult-like syntactic representation onto a proper sound representation. Lorenzo and Vares (2017, 2019) further argue that children with SLI may have more difficulty with object RCs than subject RCs because of the externalization deficit. Specifically, the relative head (in Spec CP) and the subject (in Spec TP) are in the same phase in object RCs, whereas the relative head and object are located in the CP and vP, respectively, in subject RCs. The two NPs, the relative head and the subject, are to be linearized relatively to each other at the same phase in object RCs, which will cause problems for children with SLI. This hypothesis is not flawless in that it cannot account for the prevalent mistakes observed in children with SLI in previous studies, such as the thematic role reversal errors and the omission of the relativizer.

To sum up, there is no consensus as to the source of difficulty shown in the production of RCs by children with SLI. This paper is intended to inform this debate by investigating the production of RCs in Mandarin children with SLI. The specific research questions that we will address are (1) whether or not Mandarin children with SLI differ in the production of subject and object RCs; (2) whether or not Mandarin children with SLI differ from their TD peers in the production of RCs; (3) what are avoidance strategies adopted by children with SLI when they failed to produce targeted RCs?

To date, no other studies have investigated the production of RCs in Mandarin children with SLI, hence this study could allow us to ascertain the nature of the deficits seen in them. Furthermore, we employ similar methodologies to those used in previous studies (Novogrodsky and Friedmann, 2006; Garraffa et al., 2015), and this continuity in the methodology permits us to see if the difficulty reported in children with SLI speaking other languages is evident in Mandarin-speaking children with SLI.

## Materials and Methods

### Participants

The current research included forty-three monolingual Mandarin-speaking children aged from 3; 2 to 5; 11. Both children with SLI (*N* = 13) and TD children (*N* = 30) were recruited from normal kindergartens. We asked their parents for permission to participate on behalf of all of their children.

The children with SLI were between the ages of 4;5 and 5;8 (Mean = 61.77 months; SD = 5.41 months). To choose the suspected subjects during the screening process, parents, and kindergarten teachers were required to complete a questionnaire to identify potential subjects and exclude those children who did not meet the criteria for SLI as described in Leonard (2014; p. 14–15). In fact, all our children have normal hearing ability, no otitis media with effusion, no neurological dysfunction history, no structural anomalies, no oral motor dysfunction, or no symptoms of impaired reciprocal social interaction.

During the test stage, two standardized tests were used to determine the language capacity of the suspected children with SLI. The first test is the *Peabody Picture Vocabulary Test– Revised Chinese Version 1990* (PPVT-R for short), which can be used to test Mandarin-speaking children’s receptive vocabulary with considerable validity and high reliability (Sang and Miao, 1990). The second one is *Diagnostic Receptive and Expressive Assessment of Mandarin* (DREAM for short) (Ning et al., 2014), which is considered to have potential as a diagnostic test of Mandarin language impairment for children between 2;6 and 7;11 ages (Liu et al., 2017).

The children’s performance IQ was assessed with the Wechsler Preschool and Primary Scale of Intelligence– Fourth Edition [WPPSI-IV (CN) for short] developed by King-May Psychology Assessment, Ltd., with the license from NCS Pearson, Inc. All children had non-verbal IQ within the normal range and had at least two of the six language test scores a minimum of 1 SD below their mean for age (DREAM and PPVT-R), of which the scores on the syntax in DREAM are at least 1 SD below the mean for their age. In the current study, all the children with SLI exhibit syntactic impairment, and are therefore referred to as children with Syntactic-SLI (SLI for short subsequently). Table 1 provides the individual scores for children with SLI.

**TABLE 1:** Test scores of the children with SLI.

	DREAM total	DREAM receptive	DREAM expressive	DREAM semantics	DREAM syntax	PPVT(R)
SLI 01	<−1.5 SD	<−1.5 SD	<−1.5 SD	<−1 SD	<−1.5 SD	<−1.5 SD
SLI 02	<−1 SD	≥−1 SD	<−1 SD	≥−1 SD	<−1 SD	<−1.5 SD
SLI 03	<−1.5 SD	<−1.5 SD	<−1.5 SD	<−1 SD	<−1.5 SD	<−1.5 SD
SLI 04	<−1 SD	<−1 SD	<−1 SD	≥−1 SD	<−1 SD	<−1.5 SD
SLI 05	≥−1 SD	≥−1 SD	<−1.5 SD	≥−1 SD	<−1 SD	≥−1 SD
SLI 06	≥−1 SD	≥−1 SD	<−1.5 SD	≥−1 SD	<−1 SD	<−1.5 SD
SLI 07	<−1 SD	≥−1 SD	<−1 SD	≥−1 SD	<−1 SD	<−1.5 SD
SLI 08	≥−1 SD	≥−1 SD	<−1.5 SD	≥−1 SD	<−1 SD	<−1 SD
SLI 09	≥1 SD	≥−1 SD	<−1.5 SD	≥−1 SD	<−1 SD	<−1 SD
SLI 10	<−1 SD	<−1 SD	<−1.5 SD	≥−1 SD	<−1.5 SD	<−1.5 SD
SLI 11	<−1 SD	≥−1 SD	<−1.5 SD	≥−1 SD	<−1.5 SD	≥−1 SD
SLI 12	≥−1 SD	≥−1 SD	<−1.5 SD	≥−1 SD	<−1 SD	<−1.5 SD
SLI 13	<−1 SD	<−1 SD	<−1.5 SD	<−1.5 SD	<−1 SD	<−1.5 SD

*The scores obtained from DREAM are standard scores, whereas the scores of PPVT(R) are raw scores.*

We have recruited two groups of TD children to participate in the experiment, allowing us to specify the possible discrepancy between children with and without SLI. One control group of fifteen children (Age range: 4;3–5;8; Mean = 62.1 months, SD = 4.97 months) was chosen to serve as TD children with Age Matched (TDA) and the rest were 15 younger TD children (TDY) (Age range: 3;2–4;2; Mean: 45 months, SD: 4.5 months). A one-way ANOVA test showed a significant difference among the three groups in terms of age [*F*(2,46) = 55.4, *p* < 0.01] and the *post hoc* comparisons with Bonferroni correction revealed that the TDA and SLI group do not differ in age (*MD* = −0.403, *p* = 0.832 > 0.05) and there is significant difference in age between the TDY and SLI group (*MD* = 16.76, *p* < 0.01). The TDA and TDY groups also received a standardized language test (DREAM) and their scores are within the normal range. All the TDA and TDY children are mentally and physically healthy and with normal language proficiency. Table 2 presents the age and PPVT(R) and DREAM scores of the participants.

**TABLE 2:** Detailed profiles of the three groups of children.

	AGE in months Mean (SD)	DREAM total score Mean (SD)	DREAM receptive Mean (SD)	DREAM expressive Mean (SD)	DREAM semantics Mean (SD)	DREAM syntax Mean (SD)	PPVT(R) Mean (SD)
SLI	61.77 (5.41)	85 (6.37)	87.30 (7.63)	73.38 (4.57)	90.53 (9.04)	79.30 (5.61)	24.23 (10.97)
TDA	62.18 (4.96)	114.20 (8.64)	115.33 (9.15)	107.93 (10.10)	120.86 (12.23)	107.53 (8.63)	
TDY	45.01 (4.05)	124.46 (7.09)	125.86 (7.98)	117.73 (5.10)	131.6 (9.14)	117.33 (6.95)	

*The scores obtained from DREAM are standard scores, whereas the scores of PPVT(R) are raw scores.*

### Materials

Following Novogrodsky and Friedmann (2006), we adopted a preference choice task to assess the production of RCs in Mandarin children, in which the child was required to choose one of two options presented to them. The task was designed to ensure that the response had to be formulated as a relative clause. The task consisted of twenty trials, with half eliciting subject RCs and the other half eliciting object RCs. All of the targeted subject RCs and object RCs are reversible, with the participants being always animate. The examples are given in (7).


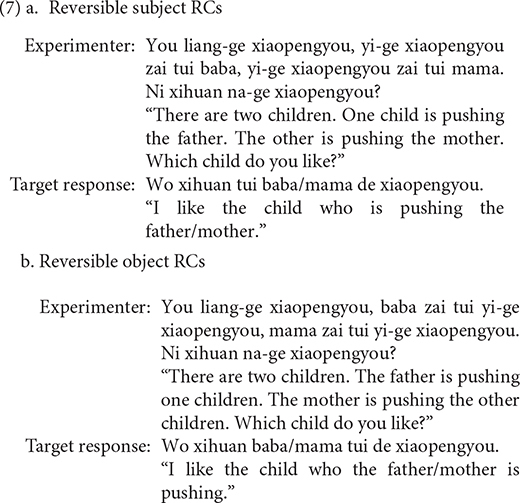


All nouns and verbs used in the task are familiar to children aged 4–6. To build up our stimuli, we used 10 transitive verbs, including *tui* “push,” *la* “hold hands,” *yao* “bite,” *bao* “hug,” *ti* “kick,” *zhua* “scratch,” *bei* “carry on back,” *qin* “kiss,” *zhuang* “bump,” and *zhui* “chase.” A total of fifteen nouns were employed to depict the animate characters (*baba* “father,” *mama* “mother,” *didi* “younger brother,” *meimei* “younger sister,” *yeye* “grandpa,” *nainai* “grandma,” *xiaomao* “cat,” *xiaogou* “dog,” *xiaozhu* “pig,” *xiaoyang* “sheep,” *xiaoniu* “calf,” *xiaolu* “deer,” *laohu* “tiger,” *shizi* “lion,” *xiaoxiong* “bear”). Experimental items were randomized and presented in the same order to all children and all of the test sentences were between nine and eleven words.

### Procedure

In a quiet room in the kindergarten, participants were tested individually in ten 30-min sessions. The RCs task and many other tasks constituted a large-scale study on Mandarin children with SLI and only the tasks relevant to this study are reported in this paper. There was one practice trail before the experiment to ensure that the participants understood the task, in which the correct answer was presented to those who failed to produce the targeted sentence. All of the elicited sentences were audio-recorded and later transcribed based on the recordings. Two researchers double-checked the coding for accuracy.

### Coding and Scoring

Various types of responses elicited were grouped into three categories: targeted RCs, non-targeted RCs, and other responses. The coding for the responses is exemplified in (8) to (17).


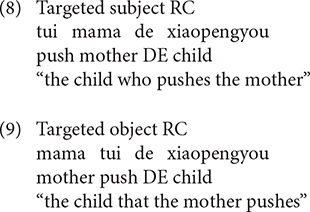


Sentences (8), (9) are targeted responses for subject RCs, object RCs, respectively. According to Liu (2005), the demonstrative *Ne* (that) can function as the relative marker. As a consequence, sentences with demonstrative *Ne* (that) as complementizer, as in (10), were also considered as target responses.


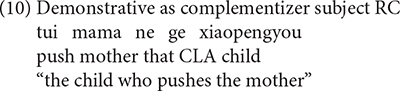


In addition, the headless RCs were also regarded as targeted responses, as exemplified in (11).


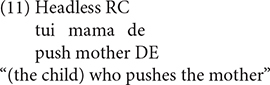


The second category of responses is called non-targeted RCs. In the object RC condition, the non-targeted RCs include passive object RCs (12), RCs with resumptive NPs (13), subject RCs with thematic role reversal (14), complementizer omission RCs (15)^4^. Although sentence (15) is identical to a declarative sentence in terms of word order, we coded it as a complementizer omission RC because the participant was required to answer the question *Ni Xihan Nage Xiaopengyou*, “which child do you like,” the children intended to complete the sentence with an NP object when they started with *Wo Xihuan* “I like.” As a consequence, it is reasonable to believe that the sentences that come after *Wo Xihuan* “I like” should be coded as a complementizer omission RC rather than a declarative sentence.


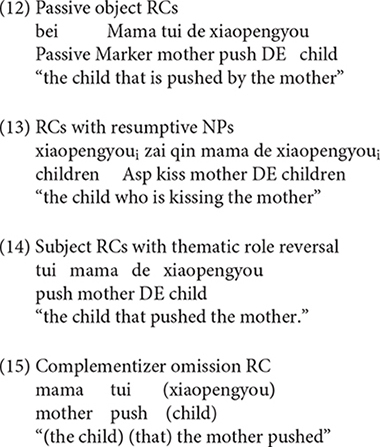


The third kind of responses are referred to as other responses, including the simple sentence (16) and sentence fragment (17), which occurred across the two conditions.


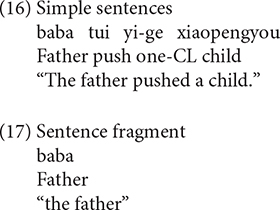


## Results

A total of 860 responses were elicited, half of them resulted from the subject RCs condition and the remainder from the object RCs condition. We used R (R Core Team, 2013) and lme4 (Bates et al., 2012) to perform a series of repeated measure logistic regression analyses in a mixed model, in which a backward elimination procedure is used to compare the goodness of fit of the models (Baayen, 2011). First, we analyze the targeted RCs responses. Table 3 displays the percentages, raw scores, means, and standard deviations of targeted RCs responses in each group, revealing a subject over object RCs advantage in all three groups of children. Notably, headless RCs account for a considerable proportion of targeted RCs responses across all three groups.

**TABLE 3:** Percentage (%), raw scores (N), means (M), and standard deviation (SD) of target responses in each group.

Groups	Subject RCs				Object RCs			
		
	%	*N*	*M*	SD	%	*N*	*M*	SD
SLI	30	39/130	3.00	3.39	18	24/130	1.84	2.26
TDA	93	139/150	9.26	1.38	64	96/150	6.85	2.44
TDY	74	111/150	7.40	2.61	48	79/150	5.26	3.45

We fitted targeted RCs responses to a mixed-effects model using sentence type (i.e., subject RCs, Object RCs) and group (SLI, TDA, TDY) as fixed factors, but the interaction between the two fixed factors was excluded in the model, and subjects and items as random factors. The results showed a significant effect of sentence type (*x^2^* = 4.05, *p* = 0.04; Wald *Z* = −2.01, *p* = 0.04), indicating that object RCs are more difficult to produce. The results also revealed a significant effect of group (*x^2^* = 10.17, *p* = 0.001; Wald *Z* = 3.91, *p* = 0.001). To be more precise, children with SLI performed worse than both TDA (*Estimate* = 5.77, *SE* = 1.01, *Wald Z* = 5.71, *p* < 0.001) and TDY children (*Estimate* = 3.06, *SE* = 0.86, *Wald Z* = 3.55, *p* < 0.001) in the production of targeted RCs, and there was a significant difference between the two groups of TD children (*Estimate* = −2.71, *SE* = 0.96, *Wald Z* = −2.80, *p* < 0.01). Thus, we can conclude that children with SLI produced significantly less targeted RCs than TDA or TDY children.

Now we turn to the analysis of non-targeted RCs responses in each of the three groups. The experiment yielded a total of 84 non-targeted RCs, as shown in Table 4. There were 7 RCs with resumptive NPs in the subject RCs condition, while the remaining 77 non-targeted RCs were collected in the object RCs condition, which included 51 subject RCs with thematic role reversal, 15 Passive object RCs, 9 Complementizer omission RCs, and 2 RCs with resumptive NPs. Complementizer omission RCs and subject RCs with thematic role reversal were the two most frequently occurring non-targeted RCs in children with SLI, but subject RCs with thematic role reversal and Passive object RCs were more prevalent in TDA children. TDY children only produced the subject RCs with thematic role reversal when an object RCs was intended.

**TABLE 4:** Numbers (N), percentage (%) (non-target RCs/all responses) of the non-target RCs in each group.

	Object RCs with thematic role reversal		Passive object RCs		Complementizer omission object RCs	
			
	*N*	%	*N*	%	*N*	%
SLI	9/130	6.9%	1/130	0.7%	9/130	6.9%
TDA	27/150	18%	14/150	9.3%	0/150	0%
TDY	15150	10%	0/150	0%	0/150	0%
Total	34/430	7.9%	15/430	3.4%	8/430	1.8%

		**Object RCs with resumptive NP**			**Subject RCs with resumptive NP**	
				
**Groups**		***N***	**%**		***N***	**%**

SLI		1/130	0.7%		4/130	3%
TDA		1/150	0.6%		2/150	1.2%
TDY		0/150	0%		1/150	0.6%

To begin, to see what influences the production of non-targeted RCs, we conducted two mixed-effects models with group as a fixed factor and subjects and items as random factors. We found there was no significant effect of group in production of subject RCs with thematic role reversal (*x^2^* = 0.016, *p* = 0.92; Wald *Z* = 0.52, *p* = 0.09) or passive object RCs (*x^2^* = 0.11, *p* = 0.73; Wald *Z* = −0.33, *p* = 0.74).

As shown in Table 4, Complementizer omission RCs were extremely rare, with only nine sentences produced by children with SLI in the object RCs condition.

We carried out the analyses with sentence type and group as fixed factors, subject and item as random factors and RCs with Resumptive NPs as a dependent variable, but the interaction between the two fixed factors was excluded in the model. The results indicated that neither group (*x^2^* = 0.10, *p* = 0.74; Wald *Z* = 0.32, *p* = 0.75) nor sentence type (*x^2^* = 0.10, *p* = 0.74; Wald *Z* = 0.33, *p* = 0.74) had a significant effect.

To investigate what children do when they fail to produce RCs, we examined other responses in our study. Table 5 presents the raw scores and percentages of other responses in each group. First, we fitted responses of simple sentences to a mixed-effects model with sentence type and group as fixed factors and subjects and items as random factors. The results demonstrated a significant effect of sentence type (*x^2^* = 7.61, *p* = 0.005; Wald *Z* = −2.76, *p* = 0.005), showing that simple sentences are more frequently produced in the object RCs condition.

**TABLE 5:** Numbers (N), percentage (%) (other responses/all responses) of other responses in each group.

Groups	Subject RCs				Object RCs			
		
	Simple sentences		Fragment		Simple sentences		Fragment	
				
	*N*	%	*N*	%	*N*	%	*N*	%
SLI	15/130	11.5%	72/130	57.6%	15/130	11.5%	75/130	57.6%
TDA	5/150	3.3%	4/150	2.6%	9/150	6%	3	2%
TDY	24/150	16%	14/150	9.3%	44/150	29%	12/150	8%

Second, we used a mixed-effects model with sentence type and group as fixed factors and subjects and items as random factors to analyze the production of fragments, excluding the interaction between the two fixed factors. The results revealed a significant effect of group (*x^2^* = 58.69, *p* < 0.001; Wald *Z* = −2.42, *p* < 0.001) and a significant effect of sentence type (*x^2^* = 24.79, *p* < 0.001; Wald *Z* = 4.94, *p* < 0.001). Children with SLI produced more fragments than TDA children (*Estimate* = −6.28, *SE* = 0.001, *Wald Z* = −5.62, *p* < 0.05). However, there was no difference between children with SLI and TDY children (*Estimate* = −4.93, *SE* = 0.001, *Wald Z* = −4.41, *p* = 0.18).

To summarize, our participants displayed a preference for subject RCs in production and children with SLI produced targeted RCs significantly less than their TD peers. When children with SLI failed to produce targeted RCs, they were liable to produce untargeted RCs such as Complementizer omission RCs and subject RCs with thematic role reversal, as well as simple sentences and sentence fragments.

## Discussion

This section will present an explanation of the findings reported in the previous section. First, we will account for the subject over object RC advantage observed in our participants and then proceed to explore the nature of the deficiency in children with SLI.

The results of this paper reveal that children with SLI performed better in the subject RCs than in the object RCs condition. The question that arises at this point is which theory might provide a plausible explanation for the asymmetry. According to the Linear Distance Hypothesis, it is the distance between the head and the gap in RCs that incurs the subject-object RCs asymmetry (Gibson, 1998, 2000 among many others). In English subject RCs, only one element intervenes between the head and the gap (the complementizer *that*), as shown in (18a), on the other hand, three elements intervene between them in object RCs (the complementizer *that*; the NP *the father* and the verb *kiss*), as shown in (18b). Object RCs are, therefore, more difficult to process than subject RCs due to the greater distance between the head and the gap.


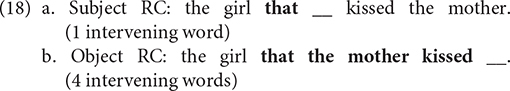


This hypothesis can not, however, account for the findings of this paper. Mandarin is a language with head-final RCs, meaning that subject RCs have more intervening words than object RCs, as shown in (19). This hypothesis predicts that Mandarin subject RCs are more difficult to produce, which contradicts our findings.


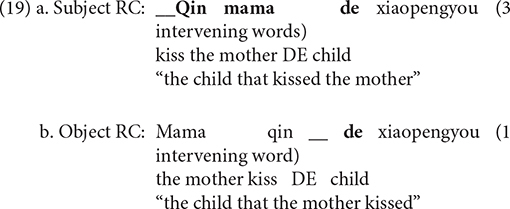


The second theory is known as the Canonical Word Order Hypothesis, which claims that the similarity or dissimilarity to canonical word order can assist or hinder the production of RCs (Diessel and Tomasello, 2000, 2005, among many others). More specifically, the complexity of English object RCs is related to non-canonical word order because they have a non-canonical word order, i.e., SOV, while subject RCs have a word order similar to simple sentences, as seen in (20).


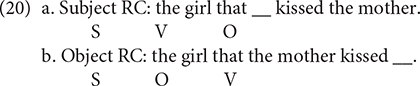


This Hypothesis also predicts that Mandarin object RCs are easier to produce than subject RCs, given that Mandarin object RCs have a word order similar to simple sentences while subject RCs have a non-canonical word order, as in (21).


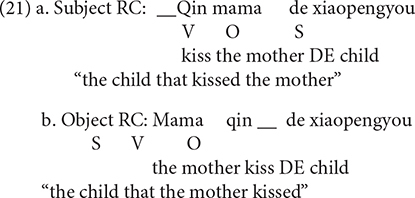


O’Grady (1997) put forward the Structural Distance Hypothesis, arguing that the complexity of RCs depends on the structural distance between the head and the gap rather than the linear distance, which is determined by the number of XP nodes intervening between the head and the gap. It is self-evident that there are less nodes between the head and the gap in Mandarin subject RCs (21a) than that in object RCs (21b), as shown in (22). According to this theory, the gap in the object RC is more deeply embedded within the syntactic tree, making it more difficult for children to produce, which has been corroborated by our findings.

However, this hypothesis cannot adequately account for all of the findings in this paper. First, it is unable to explain why the production of RCs is not asymmetric in TDA children. Second, as we will discuss in the near future, it fails to justify the participants’ avoidance strategies when they failed to produce targeted RCs.


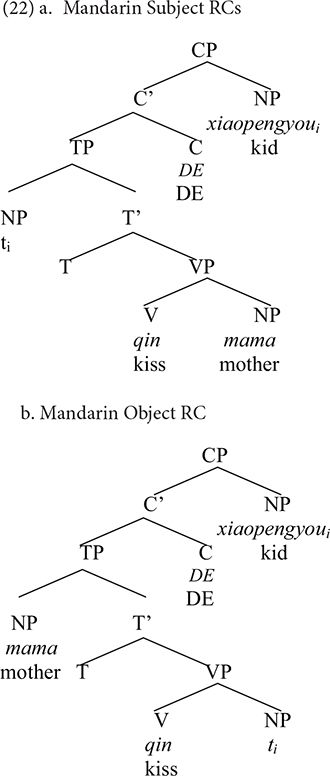


The fourth theory is the Frequency Hypothesis (Ambridge et al., 2015), assuming that the frequency with which particular patterns occur in inputs is often correlated with ease of acquisition. However, corpus studies on the input frequency of Mandarin children have yielded mixed results. Several studies have shown that subject RCs are more frequent than object RCs (Hsiao and Gibson, 2003; Pu, 2007; Wu et al., 2010). On the other hand, Chen and Shirai (2015) found that subject RCs in the input of Mandarin children are less than object RCs. Given the mixed results, we cannot determine whether our findings can corroborate the prediction of the Frequency Hypothesis.

The Semantic Prominence Hypothesis (O’Grady, 2011; p. 20) proposes that the more prominent a nominal’s referent is within an RC, the easier it is for the processor to create an aboutness relationship with it. Since a referent functioning as a subject is more prominent than a referent functioning as a direct object, the subject RC is easier to construe. Although this prediction has been borne out by our findings, this theory has also failed to account for the avoidance strategies employed by the participants in this paper.

The last one is the Relativized Minimality (RM), which is postulated as a theory of syntactic locality on constraints governing extraction from syntactic islands (Rizzi, 1990, 2004). To be more specific, in the configuration of (23), a local relation between X and Y cannot hold if the intervener Z is similar in structure to X.


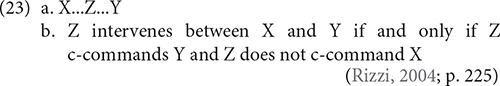


Friedmann et al. (2009) proposed that it would be difficult to establish a dependency between a relative head and its copy for young children if there is a qualified element intervening between them. In the Mandarin subject RC (22a), there is no intervener between the head (specifier of CP) and the copy (place of extraction), while the subject is an intervener between the target and the copy in the object RC (22b).

According to Friedmann et al. (2009), in the adult grammar, an intervener can block the establishment of a local A’ relation only when the intervener and the attractor share the same featural specification, as in configuration (24).


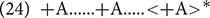


If the attractor is more richly specified in formal features than the intervener, there is no blocking effect, as in configuration (25).


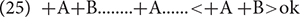


The Mandarin object RC is a case of (25), shown in (26). In this case, the attractor has a [+Rel] feature, which makes the attractor more richly specified. Thus, in adult system, (26) is ruled in.


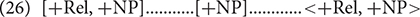


However, as proposed by Friedmann et al. (2009) and Jensen de López et al. (2014), due to their limited processing ability, young TD children and children with SLI are vulnerable to the intervention effect in (26) when the head and the copy share a structural similarity, resulting in difficulty with processing object RCs. The findings of this paper can be explained using the RM approach, but this approach does not explain why children with SLI opted for fragments and simple sentences over targeted RCs.

At this juncture, we propose that the Edge Feature Underspecification Hypothesis (Yu, 2018; p. 28–33) can better explain the findings of this paper. The hypothesis is dubbed as Edge Feature Underspecification Hypothesis (EFUH), as formulated below in (27).


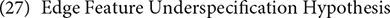


The representational deficit in children with SLI is located in the underspecified Edge Feature, and the defective Edge Feature further induces RM effect and impaired knowledge concerning the functional category.

In Generative Grammar, Edge features (EF) refer to features at the edge of the clausal domain. Chomsky (2008) has suggested that wh-movement is driven by an Edge Feature on C, which attracts a wh-expression to move to the edge of CP to become the specifier of C. It is worthy of note that the EFs on C are also known as syntactic-discourse features located in the left periphery of clauses, i.e., on the edge of CP (Rizzi, 2006), such as Questions, Topic, Focus, Relatives, etc.

The central idea of this hypothesis is that the deficit in children with SLI is caused by underspecification of EFs, and that there is significant variation in the underspecification. In line with Grillo (2009), we employ the term underspecification to characterize the impairment of children with SLI in representing the morphosyntactic featural make-up. To be more specific, we propose that underspecification of features means that children with SLI cannot properly represent the relevant features or are insensitive to them. Radford et al. (2009; p. 361) claim that when features in functional categories are underspecified, the functional categories become null and children have no suitable overt lexical item to fill the relevant slots. One of the consequences of this definition of underspecification is the total absence of functional words, which has not been corroborated by many researchers (e.g., Leonard, 1995). In contrary to their assumption, we maintain that the underspecification of features does not necessarily imply the absence of the grammatical rules governing the feature marking, but that it will result in poor performance in the acquisition of relevant constructions.

We propose that there is significant variation in underspecification and that different degrees of underspecification may result in diverse patterns of disruption and impairment on the basis of both empirical and theoretical evidence. First, this assumption is well-grounded in empirical studies. For example, Adani et al. (2016) examined the production of RCs in German-speaking children with SLI (4;7–10;11) and found that while young children with SLI encounter substantial difficulty producing RCs in general, the *wh*-movement is not absent in these children. They concluded that although *Wh*-movement is hard for this population, they did show some ability to produce adult-like sentences derived by *Wh*-movement.

Theoretically, as we will discuss shortly, we maintain that the underspecification of EFs is caused by limitations in internal syntactic processing capacities in children with SLI. More specifically, the underspecification of EFs occurs when the cost of activating them exceeds children’s processing capabilities, resulting in the features not being activated in time for successful integration into the syntactic structure. Leonard (2014, p. 271) also argued that if information is not processed quickly enough, it will succumb to faster decay or interference from the subsequent information. If the preceding is correct, we can naturally conclude that there is variation in the underspecification of EFs, as internal syntactic processing capacities vary according to a variety of conditions.

There are two reasons to propose that grammars of children with SLI operate on the basis of defective EFs. First, the EFs are encoded at the highest level of the clause (see Belletti, 2004 among many others), which is vulnerable to impairment. Second, limitations in internal syntactic processing capacities are supposed to underlie the underspecified EFs in children with SLI. The less efficient processing of syntactic information will result in the desynchronization of parts of the syntactic tree (cf., Herman, 1998; Grillo, 2008). Kail (1994) presented the Generalized Slowing Hypothesis, which asserts that children with SLI process information more slowly than TD children of the same age across all processing tasks. As a result, it is a reasonable assumption that they will have difficulty processing syntactic information retrieved later in the syntactic tree, such as EFs. We hypothesize that there is a selective impoverishment of the featural make-up of syntactic elements in children with SLI, because different types of features are accessed at different points during sentence processing (Grillo, 2008, p. 53). Because EFs are positioned at the top of clauses and thus available later in representation, it follows naturally that children with SLI’s impaired syntactic processing capacity will have a greater impact on the representation of EFs. As a result of the problematic representation of syntactic information, desynchronization of parts of the syntactic tree will occur (cf., Herman, 1998; Grillo, 2008). To be more specific, the integration of EFs into constructed syntactic constituents will be slowed down. As a result of the preceding, we propose that children with SLI will have difficulty synchronizing EFs in well-formed syntactic constituents while processing complex structures, and hence will be unable to fully represent EFs. In summary, we argue that because EFs are accessed later in the processing of complex structures, they are more likely to be compromised in an impaired syntactic system.

According to EFUH, since the [Rel] feature of the moved element is underspecified in the grammar of children with SLI, the blocking effect arises in object RCs, resulting in the subject over object RCs advantage in production. Grillo (2009) proposed that aphasics are insensitive to the edge feature of strong phases (CP and vP), causing underspecification of the feature set of elements moved to the specifier position of the strong phase. According to EFUH, the same can be extended to the case of children with SLI. This group of children are insensitive to the [+Rel] feature of the moved element, so they will assume that the target and the intervener share a structural similarity. Because of the intervener, the local dependency between the head and the copy cannot be maintained. EFUH can provide a satisfactory explanation for the subject over object RCs asymmetry in the production of RCs.

Both EFUH and standard RM account predict a subject RCs preference in children with SLI. However, the key distinction between them is that EFUH stated unequivocally that the RM effect in object RCs is caused by the underspecification of EFs in children with SLI. The normal RM account attributes the RM effect to limited computational resources for computing a subset-superset relation in young children and children with SLI (Friedmann et al., 2009; Jensen de López et al., 2014).

The avoidance strategy adopted by our participants may lend credence to the existence of the intervention effect. First, as reported previously, headless RCs, also referred to as free RCs, account for a large proportion of RC production in all three groups. We assume, based on Grosu’s (2003) analysis of Hebrew free RCs, that Mandarin free RCs are construed with a null relative operator corresponding to the Wh element. What has been moved in free RCs is a pure Wh operator that lacks a lexical NP and lacks the feature [N]. Since the subject (intervener) in the object RC has the feature [N], while the moved element has the feature [Q], TD children did not need to compute the subset–superset relationship. As a result, the production of free RCs is less taxing on the TD children. Children with SLI will also find the production of free RCs less demanding because even if the [Q] is underspecified, they will not assume the target and the intervener share a structural similarity.

The intervention effect is complex because Mandarin is an SVO language with head-final RCs. The structural intervention occurs in object RCs while there is a linear blocking effect in subject RCs. A large proportion of free RCs were obtained in the subject RC condition, indicating that the linear intervention may also affect the production of RCs. The findings are in line with the proposal from Franck et al. (2006), who discovered that linear intervention caused the production of agreement errors in a study on the production of structures involving subject-verb agreement.

Second, this study also discovered that our participants preferred subject RCs with reversed thematic roles over targeted object RCs. This avoidance strategy has also been attributed to the RM effect (Jensen de López et al., 2014). Subject RCs are easier to produce because there is no blocking element between the moved element and its copy.

Another point to note is that TDA children opted for passive object RCs instead of targeted object RCs, whereas children with SLI and TDY children almost never produced such RCs. The possible reason is that the acquisition of passives was beyond the reach of TDY children (Hirsch and Wexler, 2006) and children with SLI (van der Lely, 1996). A question arising at this point is why the TDA children used passive object RCs rather than targeted object RCs. According to Belletti (2009) among others, the presence of the subject DP in the object RC prevents the establishment of a local relationship, as seen in the configuration (28), but the RM effect can be eliminated in passive object RCs based on Collins’ (2005) “smuggling approach” to Passive derivation.


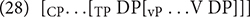


According to this approach, the verb and direct object in the passive construction move together first beyond the position of the subject and then the direct object to become the head of the RC, thus avoiding the RM violation, as shown in (29).


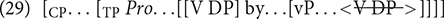


Next, we turn to the question of what is the source of the impairment observed in children with SLI. As discussed previously, some researchers assume that the lack of relativization in the narrow syntax of children with SLI is the source of difficulty shown in RCs production (e.g., Stavrakaki, 2002 among many others). However, critics have argued that the Wh-movement is not completely absent in children with SLI (Adani et al., 2016). Furthermore, the results of this paper do not corroborate Stavrakaki’s proposal given that children with SLI produced some targeted RCs (subject RCs: 30%; object RCs: 18%). Another group of researchers (e.g., Novogrodsky and Friedmann, 2006) asserts that children with SLI have impaired thematic role assignment ability. However, we discovered that this weakness is not unique to this population, as TD children have often made thematic role reversal errors^5^, suggesting that this deficit does not indicate a qualitative difference between children with and without SLI. Additionally, what this theory fails to explain is why children with SLI opt for the strategy of producing subject RCs with role reversal to take the place of targeted object RCs. To summarize, no previous SLI theory has been able to adequately account for the source of difficulty shown in the production of Mandarin RCs.

The EFUH best captures the difficulty of producing RCs by Mandarin children with SLI. According to the EFUH, children with SLI can not specify the EFs, and this underspecification results in the RM effect and structural building-up error. EFUH assumes that children with SLI have EFs that are underspecified, and thus are insensitive to the EFs of both C and the relative head. The RM effect is caused by insensitivity to EFs of relative head, while the underspecification of EFs of C causes errors concerning functional category C. To be more precise, we argue that the impairment of knowledge concerning functional categories causes the difficulty in projecting full-fledged RCs.

To begin with, children with SLI did make structural errors and omitted the embedding marker in 9 of their responses (6.9%) in the object RCs condition, whereas the TD children almost never did. The fact that the SLI children committed errors concerning the complementizer directly indicated that there is a deficiency with grammatical knowledge when it comes to the Complementizer.

Second, this paper found that when SLI children struggled to produce target RCs, they adopted declarative sentences and sentence fragments as their primary avoidance strategies, indicating that children with SLI encountered severe difficulty projecting fully fledged RCs according to EFUH. Stavrakaki (2002) assumed that syntactic knowledge in children with SLI is altogether impaired, and the production of simple declarative sentences in the RC elicitation task might be the result of controlled process. However, we maintain that the underspecification of EFs does not mean the absence of such features and the complete impaired knowledge of relativization.

Children with SLI can produce some targeted RCs according to the findings of the current study, though their ability is quite limited in comparison to their TD peers. As a result, there is no reason to claim that the use of declarative sentences and sentence fragments as avoidance strategies indicates a total lack of relativization in children with SLI. We maintain that children with SLI adopt these strategies because they can not fully specify the EF of C, resulting in the insensitivity to the semantic role of RCs.

Restrictive RCs are traditionally considered as predicates according to Quine (1960) (quoted in Heim and Kratzer, 1998) and function to restrict the referent set given in the context, similar to noun modifiers like adjectival phrases and prepositional phrases. Kuno (1976) also argued that RCs must be about the referent of their head noun, describing a property of the head of RCs. In our experiment, children with SLI were required to respond to a question with an RC to answer the question, but instead produced declarative sentences and sentence fragments. It is very likely that they are insensitive to the semantic function of RCs and consequently assume that declarative sentences and sentence fragments will function similarly to RCs. What is critical is that such insensitivity to the semantic function of RCs is consistent with EFUH’s prediction. According to EFUH, children with SLI can not specify the EF on the head of RCs, and it follows naturally that they are insensitive to the semantic feature of whole RCs.

The preceding argument is supported by a study of RC comprehension in Mandarin children with SLI (Yu, 2018; p. 107–133). The researcher found that Mandarin children with SLI made Middle errors in a character-picture matching task. Following Adani (2011), he held that the occurrence of the Middle error is an indication of a genuine problem in deriving the correct representation of RCs. In other words, committing the Middle error is equivalent to interpreting only the embedded IP of RCs. We further propose that Middle errors in comprehension are analogous to omitting the obligatory complementizer in production and the use of declarative sentences and sentence fragments, both of which are caused by deficiency of the functional category C.

To summarize, this study discovered that Mandarin children with SLI had a subject over object RC advantage in production, and that their knowledge of RCs was seriously impaired. The EFUH can explain not only the subject-object RCs asymmetry, but also the nature of errors and avoidance strategies. Theoretically, this study shows that the EFUH captures more characteristics of children with SLI in producing RCs than previous theories. Practically, this study also establishes that RC production may be clinical markers of linguistic impairment, allowing children with SLI to be distinguished from their TD peers.

## Data Availability Statement

The raw data supporting the conclusions of this article will be made available by the authors, without undue reservation.

## Ethics Statement

The studies involving human participants were reviewed and approved by the Henan Normal University. Written informed consent to participate in this study was provided by the participants’ legal guardian/next of kin.

## Author Contributions

HW and HY contributed equally to this study, including concept, experimental design, data collection, supervision, data analysis, and writing. Both authors contributed to the article and approved the submitted version.

## Conflict of Interest

The authors declare that the research was conducted in the absence of any commercial or financial relationships that could be construed as a potential conflict of interest.

## Publisher’s Note

All claims expressed in this article are solely those of the authors and do not necessarily represent those of their affiliated organizations, or those of the publisher, the editors and the reviewers. Any product that may be evaluated in this article, or claim that may be made by its manufacturer, is not guaranteed or endorsed by the publisher.

## Footnotes

1 The children produced 34 (18.6%) subject RCs and 78 (61.5%) object RCs.

2 Note that in Mandarin, “ne” functions similarly to the English pronoun “that,” but not similarly to the complementizer “that.”

3 Besides the head movement analysis of RCs, another movement analysis has been given in literature, which is referred to as operator movement analysis (e.g., Ning, 1993). According to this analysis, the head of the RC is base-generated, and the RC adjoins the head. The co-indexation between the head and the trace inside the RC is achieved through the relative operator.

4 The intended RC for (12), (14), and (15) is the object RC in (9).

5 The TDA and TDY children produced 18% and 10% subject RCs with thematic role reversal errors in the object RC condition, respectively.
